# Manipulation of the Plant Host by the Geminivirus AC2/C2 Protein, a Central Player in the Infection Cycle

**DOI:** 10.3389/fpls.2020.00591

**Published:** 2020-05-19

**Authors:** Jennifer Guerrero, Elizabeth Regedanz, Liu Lu, Jianhua Ruan, David M. Bisaro, Garry Sunter

**Affiliations:** ^1^Department of Biology, South Texas Center for Emerging Infectious Diseases, University of Texas at San Antonio, San Antonio, TX, United States; ^2^Department of Molecular Genetics, Center for Applied Plant Sciences, Center for RNA Biology, Infectious Diseases Institute, The Ohio State University, Columbus, OH, United States; ^3^Department of Computer Science, North Dakota State University, Fargo, ND, United States; ^4^Department of Computer Science, University of Texas at San Antonio, San Antonio, TX, United States

**Keywords:** AC2/C2, pathogenicity factor, transcriptional activation, PTGS, TGS, antiviral defense response

## Abstract

Geminiviruses are a significant group of emergent plant DNA viruses causing devastating diseases in food crops worldwide, including the Southern United States, Central America and the Caribbean. Crop failure due to geminivirus-related disease can be as high as 100%. Improved global transportation has enhanced the spread of geminiviruses and their vectors, supporting the emergence of new, more virulent recombinant strains. With limited coding capacity, geminiviruses encode multifunctional proteins, including the *AC2/C2* gene that plays a central role in the viral replication-cycle through suppression of host defenses and transcriptional regulation of the late viral genes. The AC2/C2 proteins encoded by mono- and bipartite geminiviruses and the curtovirus C2 can be considered virulence factors, and are known to interact with both basal and inducible systems. This review highlights the role of AC2/C2 in affecting the jasmonic acid and salicylic acid (JA and SA) pathways, the ubiquitin/proteasome system (UPS), and RNA silencing pathways. In addition to suppressing host defenses, AC2/C2 play a critical role in regulating expression of the coat protein during the viral life cycle. It is important that the timing of CP expression is regulated to ensure that ssDNA is converted to dsDNA early during an infection and is sequestered late in the infection. How AC2 interacts with host transcription factors to regulate CP expression is discussed along with how computational approaches can help identify critical host networks targeted by geminivirus AC2 proteins. Thus, the role of AC2/C2 in the viral life-cycle is to prevent the host from mounting an efficient defense response to geminivirus infection and to ensure maximal amplification and encapsidation of the viral genome.

## Introduction

**FIGURE 1:**
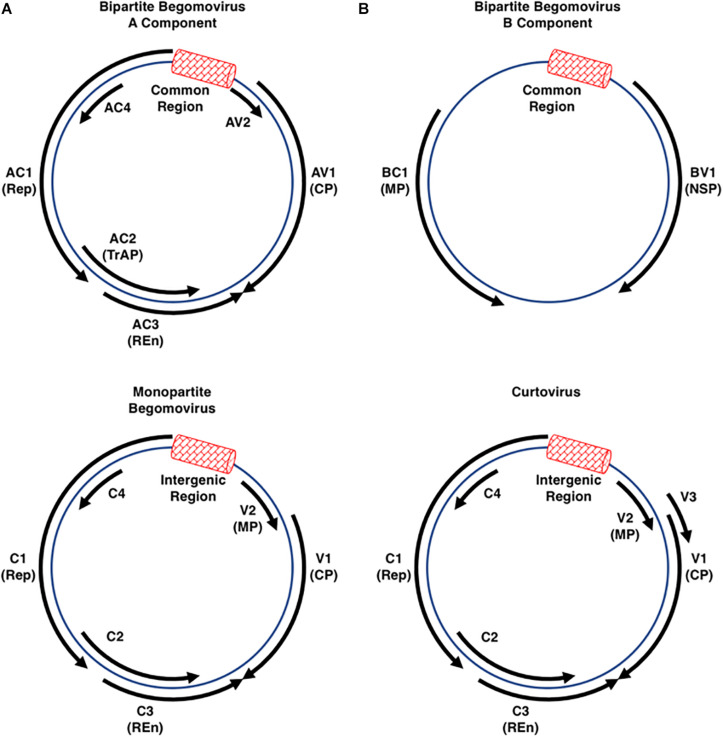
Begomo- and Curtovirus Genome Organization. The circular diagrams represent the double-stranded replicative forms of geminiviruses belonging to the genera *Begomovirus* (mono- or bipartite genomes) and *Curtovirus* (monopartite genomes only). Each has an intergenic region (IR: red box) that contains the origin of rolling-circle replication flanked by divergent promoters that control expression of the virion and complementary sense transcription units. Viruses belonging to each of these genera (see Table 1 for a list of viruses discussed in this review) encode between six and eight open reading frames (solid black arrows) capable of encoding proteins larger than 10 kDa. The coat protein (CP) forms the viral capsid that mediates vector transmission, and in monopartite viruses also functions as a movement protein. In the monopartite begomoviruses and curtoviruses, the AV2/V2 proteins inhibit gene silencing and also function as movement proteins (MP). The AC1/C1 protein (Replication initiator protein-Rep) is absolutely required for initiation of viral DNA replication, while the AC3/C3 replication enhancer protein (REn) stimulates replication. The AC2 protein in bipartite and monopartite begomoviruses is also called transcriptional activator protein (TrAP) due to its function in transcriptional activation of the CP and, in bipartite viruses, the BV1 gene. AC2 also interferes with transcriptional gene silencing (TGS) and post-transcriptional gene silencing (PTGS), functions it shares with the related C2 protein of the monopartite begomoviruses and curtoviruses. The AC4/C4 protein has been shown to function as a suppressor of PTGS in some, but not all viruses. In bipartite begomoviruses the movement proteins BV1 (Nuclear shuttle protein, NSP) and BC1 (MP) are encoded by DNA-B.

The *Geminiviridae* is a family of single-stranded DNA (ssDNA) viruses that infect agricultural crops in tropical and sub-tropical regions worldwide, and are responsible for billions of dollars in annual losses contributing to famine and loss of life (Legg and Fauquet, 2004; Scholthof et al., 2011). The International Committee on Taxonomy of Viruses (ICTV) currently recognizes nine genera within the family, classified according to genome organization, genome-wide pairwise sequence identities, insect vector, and host range (Zerbini et al., 2017; Kumar, 2019). The genus *Begomovirus*, with ∼320 species, is by far the largest and its members are the most widely studied (Zerbini et al., 2017; Kumar, 2019). By contrast, the genus *Mastrevirus* consists of ∼30 species, with the remaining genera (*Becurtovirus*, *Capulavirus*, *Curtovirus*, *Eragrovirus*, *Grablovirus*, *Topocuvirus*, and *Turncurtovirus*) having 1–4 species each (Zerbini et al., 2017). Geminiviruses do not encode DNA or RNA polymerases and rely on host machinery to replicate their circular ssDNA genomes through double-stranded DNA (dsDNA) replicative forms (RFs). The RFs associate with host histone proteins to form non-integrating minichromosomes. Most geminiviruses have monopartite genomes except for the begomoviruses, which have either monopartite or bipartite genomes (designated DNA-A and DNA-B), both of which are required for infectivity. Individual genome components of bipartite begomoviruses are typically 2.5–3.0 kb in size and together can encode a total of eight proteins, while monopartite begomovirus genomes are ∼3.0 kb and encode six proteins. Curtovirus genomes, also ∼3.0 kb, are similar in organization to monopartite begomoviruses and encode seven proteins (Figure 1). Considerable genetic economy is evident, with genes encoded by both strands of the dsDNA RFs and overlapping genes in different reading frames. Two gene nomenclature systems are in use. One denotes genes and proteins as leftward (L) or rightward (R) relative to conventional genome maps. The other refers to genes as complementary (C) or viral (V) sense, with viral sense indicating the encapsidated strand. The latter system is used in this review. Most viral proteins are also named according to core function.

**TABLE 1:** Geminiviruses discussed in this review.

Bipartite begomovirus	Abbreviation	Monopartite begomovirus	Abbreviation	Curtovirus	Abbreviation
*African cassava mosaic virus*	ACMV	*Cotton leaf curl Kokhran virus*	CLCuKoV	*Beet curly top virus*	BCTV
*Bean dwarf mosaic virus*	BDMV	*Cotton leaf curl Multan virus*	CLCuMuV	*Beet curly top virus-SpCT*	BCTV-SpCT
*Cabbage leaf curl virus*	CaLCuV	*Papaya leaf curl virus*	PaLCuV	*Beet severe curly top virus*	BSCTV
*Mungbean yellow mosaic virus-Vigna*	MYMV	*Tomato yellow leaf curl China virus*	TYLCCNV		
*Tomato golden mosaic virus*	TGMV	*Tomato yellow leaf curl China betasatellite*	TYLCCNB		
*Tomato leaf curl New Delhi virus*	ToLCNDV	*Tomato leaf curl Java virus*	TLCJV		
		*Tomato yellow leaf curl-Sardinia virus*	TYLCSV		
		*Tomato yellow leaf curl virus*	TYLCV		

*Geminiviruses mentioned in this review are listed according to genus and, in the case of begomoviruses, genome type (bipartite or monopartite).*

In all geminiviruses, virion and complementary sense genes are separated by an intergenic region (IR) ∼300 bp in size, a portion of which, called the Common Region (CR), is shared by DNA-A and DNA-B in bipartite begomoviruses. Transcription occurs bidirectionally from promoters within the IR, which also contains the origin of replication. In bipartite begomoviruses, the virion sense strand of DNA-A encodes the capsid protein (CP, also known AV1). In monopartite begomoviruses, the virion sense strand encodes CP/V1 that also acts as a movement factor, as well as an additional movement protein (MP/V2). In all begomoviruses, the complementary strand encodes the replication protein (Rep, AC1/C1), a transcriptional activator protein and pathogenicity factor (TrAP, AC2/C2), a replication enhancer protein (REn, AC3/C3), and AC4/C4, which also appears to function as a pathogenicity determinant. In some begomoviruses, including *African cassava mosaic virus* (ACMV), *Bean golden mosaic virus* (BGMV), *Potato yellow mosaic virus* (PYMV), and *Tomato golden mosaic virus* (TGMV), the *AC4* gene is not critical for virus infection (Elmer et al., 1988; Etessami et al., 1991; Sung and Coutts, 1995; Hoogstraten et al., 1996; Bull et al., 2007). DNA-B of bipartite begomoviruses codes for two proteins, a nuclear shuttle protein (NSP, BV1) in the virion sense, and a movement protein (MP, BC1) in the complementary sense (Stanley and Gay, 1983; Hamilton et al., 1984; Zerbini et al., 2017). In the similarly organized genomes of curtoviruses, the complementary sense strand also codes for Rep/C1, C2, REn/C3, and C4. Rep and REn are highly conserved between the two genera, and the REn proteins are functionally interchangeable (Hormuzdi and Bisaro, 1995). However, while begomovirus TrAP/AC2/C2 and curtovirus C2 share some pathogenicity functions, curtovirus C2 is not a transcriptional activator (Sunter et al., 1994; Baliji et al., 2007). The curtovirus virion sense strand codes for the capsid protein which also functions as a movement protein (CP/V1), a protein that regulates viral ssDNA versus dsDNA levels (V2), and a protein required for systemic spread (V3) (Hormuzdi and Bisaro, 1993, 1995; Baliji et al., 2004).

## Geminivirus AC2/C2 Protein and the Viral Replication Cycle

Geminiviruses exhibit a strategy that is common among DNA viruses, where gene expression is separated into early and late phases. Genes that are expressed early, meaning prior to DNA synthesis, typically encode proteins necessary for replication of the viral genome and/or to modulate the host cell environment. After viral DNA replication, the late genes encoding structural proteins needed to package DNA and form virions are expressed. DNA tumor viruses, including polyomaviruses such as SV40 and papilloma viruses, rely on cellular polymerases that are typically expressed during S-phase of the cell cycle. Thus, they have evolved mechanisms to subvert many of the cellular checkpoints that control cell cycle. Geminiviruses have also evolved to produce a protein, Rep, that interferes with cell cycle controls to drive infected cells from quiescence into S-phase in order to promote virus replication (for review see Hanley-Bowdoin et al., 2013). The viral proteins that interfere with cell cycle control, including geminivirus Rep protein, also autoregulate their own expression (Sunter et al., 1993; Eagle et al., 1994). As an example, when the large T-antigen of SV40 reaches a threshold concentration it binds to the early promoter, repressing initiation of transcription (Tjian, 1981). In many cases, products of the viral early genes are also required for expression of the late viral genes, and for suppression of host defenses. We suggest that AC2 protein of geminiviruses provides this critical function in the switch from the early phases of infection, namely transformation of cells to promote viral replication, to the late phases of infection involving virion production. The focus of this review is therefore on the AC2 protein of begomoviruses and the C2 protein of monopartite begomoviruses and curtoviruses, and highlights the central role that AC2 plays in the viral replication cycle through suppression of host defenses and transcriptional regulation of the late viral genes.

## Features of the AC2/C2 Protein

The *Begomovirus* AC2 protein (also known as AL2 and Transcriptional Activator Protein; TrAP) is ∼15 kDa in size and is conserved among all Begomoviruses. Using the protein of TGMV as a representative, full-length AC2 comprises 129 amino acids with a basic N terminal region (amino acids 13 to 28), a nuclear localization signal (NLS; amino acids 17 to 31), and a C terminal acidic region (amino acids 101 to 129) containing a transcriptional activation domain (TAD, amino acids 115 to 129) (Hartitz et al., 1999).

**FIGURE 2:**
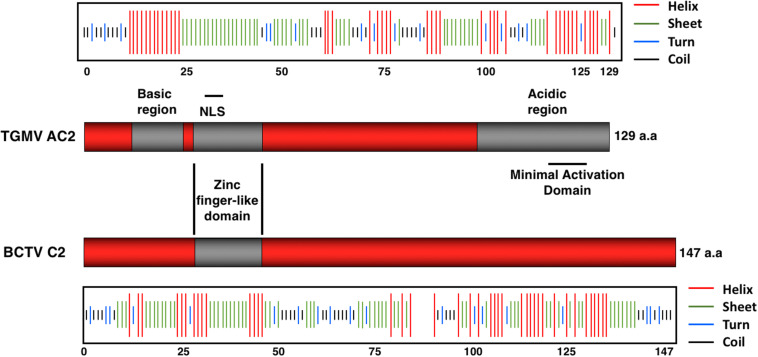
Structural Features of the AC2 and C2 Proteins. The TGMV AC2 protein is 129 amino acids in length and contains three domains: an N-terminal basic region, a zinc-finger-like domain (ZFD) that contains a nuclear localization signal (NLS), and a C-terminal acidic region that contains a minimal transcriptional activation domain. The BCTV C2 protein is 147 amino acids in length and exhibits little similarity with TGMV AC2 other than the ZFD. Secondary structure predictions were obtained using the Network Protein Sequence @nalysis (NPS@). Stretches of amino acids capable of forming helices (red lines), sheets (green lines), turns (blue lines), and coils (black lines) are shown above (AC2) or below (C2) each protein.

The central region of AC2 contains a series of conserved cysteine and histidine residues (amino acids 33 to 56) and a zinc finger-like domain (ZFD; amino acids 36 to 53) (Figure 2). AC2 has been shown to bind zinc and to be a target for phosphorylation (Hartitz et al., 1999; Wang et al., 2003). Cell localization experiments have shown that a phosphorylated form of AC2 can be detected in the nucleus and that non-phosphorylated AC2 can be found in both the nucleus and cytoplasm (Wang et al., 2003). Interestingly, AC2 is capable of self-interaction, with AC2:AC2 complexes found primarily in the nucleus (Yang et al., 2007). The ZFD is required but not sufficient for self-interaction. In contrast, AC2 interactions with cellular factors that condition antiviral defenses occur in both the nucleus and the cytoplasm (see below). Thus, AC2 localizes to sub-cellular compartments that correlate with known functions of the protein, which are to interact with plant nuclear and cytoplasmic proteins to suppress host defenses, and to activate transcription (Hao et al., 2003; Wang et al., 2003).

The equivalent C2 protein encoded by curtoviruses (Figure 2), including *Beet curly top virus* (BCTV) and *Beet curly top virus*-SpCT (BCTV-SpCT), exhibits very little sequence similarity to the AC2 protein, apart from the central region containing the conserved cysteine and histidine residues within the zinc finger-like motif. The limited sequence and structural similarity correlate with the observation that the C2 protein in curtoviruses appears to lack the ability to activate transcription (Sunter et al., 1994). Prediction of protein structures for AC2 and C2 reveals regions of alpha helix with significant stretches of coiled and extended sheet (Combet et al., 2000), but no significant similarities to known 3D structures. The major similarity in predicted secondary structure between the AC2 protein of TGMV and the C2 protein of BCTV is apparent within the ZFD, which is the only significant stretch of amino acid similarity (Figure 2). It is likely that solving the 3D structures of AC2 and C2 would provide insight into the different functions assigned to these multifunctional viral proteins.

## AC2/C2 and Suppression of Plant Defense Responses

One of the two major roles identified to date for the AC2/C2 proteins of begomo- and curtoviruses is suppression of host immune responses. Some of the host defense systems that these proteins are known to interact with include both basal and inducible defenses. The latter include the jasmonic acid and salicylic acid (JA and SA) pathways, the ubiquitin/proteasome system (UPS), and RNA silencing pathways. The AC2/C2 proteins encoded by mono- and bipartite geminiviruses and curtovirus C2 have been recognized as pathogenicity determinants based on their capacity to cause damage in a host. Inactivating mutations in the *AC2* gene renders begomoviruses non-infectious due to loss of CP and BR1 expression (Elmer et al., 1988; Sunter et al., 1994), and curtovirus C2 mutants exhibit a recovery phenotype (Hormuzdi and Bisaro, 1995). This suggests that AC2/C2 can be considered virulence factors, based on the definition of a virulence factor as a microbial component that damages the host (Casadevall and Pirofski, 1999). In many cases, virulence factors are microbial effectors that allow the pathogen to inhibit and/or evade host immune responses. Based on the classical zigzag model for plant immunity (Jones and Dangl, 2006), the first line of defense is recognition of pathogen-associated molecular patterns (PAMP) by host pattern recognition receptors (PRRs), resulting in activation of PAMP-triggered immunity (PTI). In response, successful pathogens secrete effectors that act to suppress PTI responses, leading to effector-triggered susceptibility (ETS). As a second line of defense, plants have evolved cytoplasmic R proteins (nucleotide binding–leucine-rich repeat proteins, NB-LRR) that recognize the presence or activity of specific effectors, resulting in effector-triggered immunity (ETI) (Jones and Dangl, 2006). ETI typically leads to a hypersensitive response (HR) and systemic acquired resistance (SAR). More recently, it has been proposed that there is not really a clear distinction between PAMPs and effectors, or between PAMP receptors and resistance proteins (Thomma et al., 2011). This implies that PTI and ETI are not always distinct defense responses but both can be robust or weak, depending on the specific interaction. Therefore, activation of innate immunity in plants can be summed up by recognition of danger signals either directly derived from the microbe (PAMPs and effectors) or from damage or alteration of eukaryotic host structures (Thomma et al., 2011). This definition seems appropriate for geminivirus AC2/C2 proteins, which can be considered viral effectors essential for a productive infection that can also trigger HR in some cases. Currently, the available evidence suggests that the AC2/C2 proteins interact with several host immune pathways to evade host defense responses.

## AC2 and the Hypersensitive Response

Effector-triggered immunity against a plant pathogen is a localized resistance reaction that typically involves a hypersensitive response (HR), characterized by localized cell death that often leads to arrest of the pathogen (Durrant and Dong, 2004). HR is a widespread response which can be induced by effector proteins produced by fungi, oomycetes, bacteria, viruses, and insects (Balint-Kurti, 2019). The response is characterized by a transient burst of reactive oxygen species, strengthening of plant cell walls, and accumulation of antimicrobial phytoalexins (Dangl et al., 1996). Plants exhibiting HR can develop resistance to a secondary infection through spread of a mobile signal, salicylic acid (SA), to distal tissues, a phenomenon known as systemic acquired resistance (SAR) (Durrant and Dong, 2004). Both SA and its association with the accumulation of pathogenesis-related (PR) proteins are thought to be required for an effective SAR (Durrant and Dong, 2004).

**FIGURE 3:**
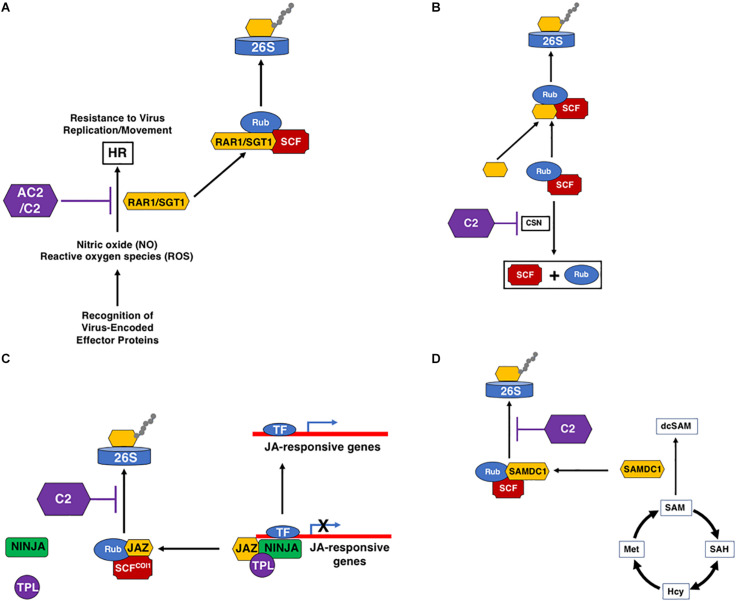
Interactions of AC2/C2 with the Ubiquitin/Proteasome System (UPS). C2 and AC2 can inhibit the UPS at multiple steps through inactivation of the CSN complex. **(A)** Inhibition of the HR response by C2 and AC2. This may be mediated through inhibition of the SCF complex that degrades RAR1/SGT1. **(B)** C2 inhibition of the CSN complex that promotes derubylation of cullin within the SCF complex. **(C)** Inhibition of SCF^COI1^ by C2, which promotes the degradation of JAZ through the 26S proteasome releasing the co-repressors TPL and NINJA. Loss of JAZ promotes the induction of JA-responsive defense genes. **(D)** Stabilization of SAMDC1 by C2 increases levels of decarboxylated SAM (dcSAM) at the expense of SAM. The following acronyms are used: HR, Hypersensitive Response; SCF, SKP1/Cullin/F-Box-; Rub, Related to Ubiquitin; RAR1/SGT1, Required for Mla12 resistance/Suppressor of G_2_ allele of Skp1; CSN, COP9 Signalosome Complex; JAZ, Jasmonate-Zim Domain; 26S, 26S Proteosome; SAM, S-Adenosyl Methionine; SAMDC1, SAM Decarboxylase 1; dcSAM, decarboxylated SAM; SAH, S-Adenosyl Homocysteine; Hcy, Homocysteine; Met, Methionine; AC2/C2, geminivirus virulence factors; TF, Transcription Factor; TPL, Topless Co-Repressor; NINJA, Novel Interactor of Jaz. Black arrows represent reactions and/or responses that are induced. Purple lines indicate inhibition through interaction with the geminivirus AC2 and/or C2 proteins.

A number of geminivirus proteins, including Rep, NSP, and V2 (Garrido-Ramirez et al., 2000; Amin et al., 2011), have been shown to induce a reaction typical of an HR response when over-expressed, indicating that these proteins could be pathogenicity determinants and a target of host immune defenses, triggering ETI. However, HR is not usually observed in plants infected with geminiviruses, suggesting that induction of an HR through recognition of some viral proteins is usually suppressed by other viral-encoded proteins (Mubin et al., 2010; Matic et al., 2016). For example, the V2 protein of several monopartite begomoviruses, including *Cotton leaf curl Kokhran virus* (CLCuKoV), *Papaya leaf curl virus* (PaLCuV), and *Tomato leaf curl Java virus* (TLCJV), is able to induce HR in *Nicotiana benthamiana* and *N. tabacum* (Mubin et al., 2010; Sharma and Ikegami, 2010). In these monopartite begomoviruses, the C2 protein is able to counter the HR response induced by the V2 protein (Mubin et al., 2010). In the case of the bipartite begomoviruses *Tomato leaf curl New Delhi virus* (ToLCNDV) and *Bean dwarf mosaic virus* (BDMV), it appears as though NSP is the inducer of HR, and the AC2 protein counteracts the HR in an NLS and ZFD-dependent manner (Hussain et al., 2007). In another example, the C2 protein of *Tomato yellow leaf curl-Sardinia virus* (TYLCSV) elicits a strong HR response in *N. benthamiana*, *N. tabacum*, and *A. thaliana*, suggesting that C2 may represent the viral effector (Matic et al., 2016). However, no HR develops during a typical TYLCSV infection, and co-expression of C2 with the Rep or V2 proteins partially counteracts the HR, resulting in chlorosis (Matic et al., 2016). Interestingly, the C2 protein from the closely related *Tomato yellow leaf curl virus* (TYLCV) does not induce HR (Matic et al., 2016). These examples suggest that geminiviruses encode proteins that are potentially recognized by the host as avirulence (*avr*) factors, resulting in development of an HR response that could inhibit viral spread. However, in some cases at least, the virus has developed countermeasures, and the AC2/C2 protein appears to have evolved to avoid or inhibit the HR response, allowing for systemic spread of the virus (Figure 3A). While this function does not appear to be unique to AC2/C2, it is clear that these proteins often play a critical role in ensuring evasion of host immune responses. It should be noted that a host protein capable of triggering an HR-like response against a geminivirus has yet to be identified.

## C2 and the Ubiquitin/Proteasome System (UPS)

Plants, and all eukaryotes, rely on a highly dynamic UPS which targets proteins for ubiquitination, a post-translational modification leading to proteolytic degradation by the proteasome [reviewed in (Dreher and Callis (2007)]. This highly enzymatic process is not only a way of regulating endogenous proteins involved in many plant cellular processes, including hormonal responses [reviewed in Santner and Estelle (2009)], but is also a defense mechanism against pathogenic organisms, including geminiviruses (Lozano-Durán et al., 2011; Mandadi and Scholthof, 2013). In plants, the most abundant family of E3 ligases comprises the multi-subunit Cullin RING Ligases (CRLs) (Dreher and Callis, 2007). Within this family, the most abundant class of SCF complexes is composed of SKP1/ASK (S-phase kinase-associated protein), Cullin1 (CUL1), an F-Box substrate binding protein, and the RING subunit RBX1 (RING box 1). The UPS appears to be a high value target for the geminiviruses (Figure 3). The AC2/C2 proteins compromise the activity of several SCF complexes, resulting in altered SCF complexes involved in plant defense and in the signaling pathways of several hormones (Lozano-Durán et al., 2011). The C2 protein of the monopartite begomoviruses TYLCSV and TYLCV, and the curtovirus BCTV, inhibit the activity of CSN5 (Figure 3B), the only catalytic subunit of the COP9 signalosome complex (CSN), but does not interfere with the assembly of CSN or SCF complexes (Lozano-Durán et al., 2011). CSN5 is necessary for CSN-mediated removal of a ubiquitin-like moiety, RUB (Related to Ubiquitin), from CUL1, which is the scaffold protein of the CRLs (Dreher and Callis, 2007). Conjugation of RUB to CUL1 upregulates CRL activity and is known to stimulate ubiquitination of substrate proteins by CRLs (Duda et al., 2008). As the CSN complex is one of the regulators of CRL activity, it is essential for function *in vivo* (Hotton and Callis, 2008), and is predicted to act as a negative regulator of SCF complexes. However, genetic data suggests that the CSN acts as a positive regulator of cullin-based SCFs and loss-of-function mutants in CSN subunits mimic mutants in SCF complexes, and result in loss of SCF activity (Cope and Deshaies, 2003). This is observed in C2-mediated inhibition of CSN5, which is predicted to result in reduced derubylation that should increase SCF activity. However, C2 actually appears to inhibit the function of CUL1-based SCF complexes resulting in altered cellular responses, including suppression of JA-responses (Lozano-Durán et al., 2011). One proposed hypothesis is SCF activity is not strictly dependent upon CSN, but that CSN is required for maintaining an optimal pool of active E3 complexes (Cope and Deshaies, 2003). If so, then CSN could act as a positive regulator of some SCF complexes and a negative regulator of others. Therefore, the CSN could be a valuable target for geminiviruses through C2, by inhibiting the activity of select SCF complexes but enhancing the activity of others.

The main SCF-dependent hormone signaling pathway impaired by C2-mediated inhibition of CSN5 is the JA response (Lozano-Durán et al., 2011). This could be particularly significant for viral pathogenesis, as many geminiviruses are limited to phloem cells, the preferential sites of JA biosynthesis, making suppression of the JA response during infection feasible (Hanley-Bowdoin et al., 2013). Plants respond to JA through degradation of the JAZ family of transcriptional regulators by SCF^COI1^ (CORONATINE INSENSITIVE 1), in a proteasome-dependent manner (Figure 3C). This suggests that the C2 protein of geminiviruses may alter the JA response by inhibiting the targeting of JAZs for ubiquitination and degradation via the 26S proteasome pathway, thereby interrupting expression of JA-responsive genes (Lozano-Durán et al., 2011; Rosas-Díaz et al., 2016). This is supported by data showing that infection of JA-treated Arabidopsis plants with BCTV resulted in milder symptoms and reduced viral DNA accumulation (Lozano-Durán et al., 2011), and that Arabidopsis plants expressing C2 from TYLCV or TYLCSV show suppression of JA-mediated defense processes and JA-dependent secondary metabolism (Rosas-Díaz et al., 2016). In addition, transcriptomic analysis of transgenic Arabidopsis plants expressing TYLCSV C2 found a subset of repressed genes in processes related to plant defense and response to JA. This is also evident in Arabidopsis plants infected with *Cabbage leaf curl virus* (CaLCuV), where COI1-induced genes are reduced, and transcripts encoding components of the ubiquitin-proteasome pathway, including 32 genes specifying 11 core and 13 regulatory subunits of the 26S proteasome complex, were elevated (Ascencio-Ibañez et al., 2008). Together, this supports repression of the JA pathway by C2, which could provide a biological advantage for viral infection through suppression of hormone-mediated plant defense responses (Lozano-Durán et al., 2011).

A second example of geminiviruses interfering with the UPS to promote virulence is exemplified by the *Beet severe curly top virus* (BSCTV) C2 protein, which interacts with and inhibits UPS-mediated degradation of *S*-adenosyl-methionine decarboxylase 1 (SAMDC1) (Zhang et al., 2011; Figure 3D). Levels of *S*-adenosyl-methionine (SAM) are modulated in part through the decarboxylase activity of SAMDC1, which therefore affects host DNA methylation status (Mandadi and Scholthof, 2013). Loss of function of either SAMDC1 or mutation in the BSCTV C2 gene leads to enhanced *de novo* methylation of the BSCTV dsDNA RF, resulting in reduced BSCTV replication and decreased BSCTV infectivity (Zhang et al., 2011). Whether the SCF complex that degrades SAMDC1 is regulated by the same CSN complex that controls SCF-dependent JA-signaling is currently unknown (Lozano-Durán et al., 2011).

An additional hypothesis has also been proposed whereby C2 may redirect certain SCF complexes to degrade specific host proteins producing an environment conducive for viral infection (Lozano-Durán et al., 2011). For example, the tobacco *N* gene–mediated resistance response against *Tobacco mosaic virus* (TMV) requires a functional RAR1/SGT1 (Required for Mla12 resistance/Suppressor of G_2_ allele of Skp1) complex (Mandadi and Scholthof, 2013). This complex physically interacts with SKP1 and the CSN (Liu et al., 2002; Figure 3). Down-regulation of components of the RAR1/SGT1, SKP1 or CSN complex abolishes *N* gene–mediated resistance, supporting a role for UPS in *N*-mediated HR and resistance responses (Liu et al., 2002). It is interesting to speculate that the ability of AC2/C2 proteins of geminiviruses to counter the HR response is a consequence of interference with the UPS and its function in antiviral immune responses. Thus, C2 interference with derubylation of the SCF complex could result in accumulation of an active SCF complex, and therefore degradation of the RAR1/SGT1 complex, preventing HR.

## Sulfur-Enhanced Defense (Sed) and C2

Based on the established link between jasmonate signaling and sulfur metabolism, it is possible that the geminivirus C2 protein targets the sulfur metabolic pathway by suppressing the JA response (Lozano-Duran et al., 2012). This could be significant given the importance of the pathway in response to plant pathogens. This is highlighted by the high response of genes related to sulfur metabolism in Arabidopsis plants treated with methyl jasmonate (MeJA), although the mechanism is still unresolved (Jost et al., 2005). Plants assimilate inorganic sulfur from the soil as sulfate which is assimilated into cysteine (Rausch and Wachter, 2005; Lozano-Duran et al., 2012). From cysteine, sulfur is available for synthesis of many different compounds, including methionine, glucosinolates, and phytoalexins as well as sulfur-containing defense compounds (SDCs) such as glutathione (Rausch and Wachter, 2005; Lozano-Duran et al., 2012). Glutathione is an important compound for protection against reactive oxygen species (ROS) that accumulate in response to stress, and so operates as a detoxification mechanism (Rausch and Wachter, 2005). Transgenic Arabidopsis plants expressing TYLCSV C2 protein that exhibit repression of genes involved in the JA response also exhibit repression of genes in the sulfur assimilation pathway (*APS3, APR1*, and *APR3*) (Lozano-Durán et al., 2011; Lozano-Duran et al., 2012). Treatment with MeJA is able to restore expression of genes involved in sulfur assimilation (Lozano-Duran et al., 2012). Thus, the interaction of the geminivirus C2 protein with proteins that function in the ubiquitination pathway appears to have a high value with respect to suppressing JA and sulfur-enhanced defense pathways. During ETI, both JA and SA, which are normally antagonistic defense hormones, accumulate to high levels, and JA appears to be a positive regulator of Resistance to *Pseudomonas syringae* 2 (RPS2)-mediated ETI (Liu et al., 2016). This is again consistent with C2 being a viral effector protein that suppresses ETI leading to effector-triggered sensitivity.

It is important to note that while the UPS appears to be a high value target for geminivirus AC2/C2 proteins, it seems unlikely that a single host protein is impacted. It is possible that geminiviruses need to enhance degradation of some host proteins and inhibit the degradation of others. Thus, a more likely scenario is that AC2/C2 target multiple proteins of the UPS system, similar to AC2/C2 mechanisms that have evolved to interfere with different components of the RNA silencing pathway (Bisaro, 2006; Raja et al., 2010).

## AC2/C2 and Metabolism

**FIGURE 4:**
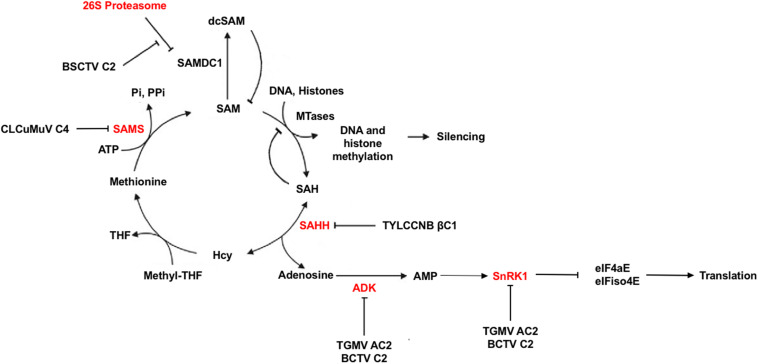
Methyl cycle Inactivation by Geminivirus and Betasatellite Proteins. S-adenosyl methionine (SAM) serves as a methyl group donor for most transmethylation reactions. The product, S-adenosyl homocysteine (SAH), is converted by S-adenosyl homocysteine hydrolase (SAHH) to homocysteine (Hcy) and adenosine. Adenosine phosphorylation by adenosine kinase (ADK) is essential because the reaction catalyzed by SAHH is reversible and the equilibrium lies in the direction of SAH synthesis. In addition, SAH is a competitive inhibitor of methyltransferase (MTase) reactions. Thus, phosphorylation of adenosine promotes flux through the cycle, and inactivation of ADK by TGMV AC2 and BCTV C2 inhibits SAM synthesis. AMP generated by adenosine phosphorylation sustains SNF1-related kinase 1 (SnRK1) activity, which also is directly inhibited by AC2 and C2. SnRK1 phosphorylation of eIF4E/iso4E interferes with protein synthesis. The TYLCCNV betasatellite TYLCCNB encodes the pathogenicity factor βC1, which blocks the methyl cycle by inhibiting SAHH activity. BSCTV C2 antagonizes proteasome-mediated degradation of SAM decarboxylase (SAMDC), increasing levels of decarboxylated SAM (dcSAM). The C4 protein of CLCuMuV interferes with SAM synthetase (SAMS) activity. Based on Yang et al., 2011.

In addition to what may be described as classical defense mechanisms, plants are capable of altering their metabolic systems in times of environmental or parasitic stress. In this regard, geminiviruses have been shown to target and inactivate two proteins important for plant cellular metabolism, sucrose non-fermenting-related kinase 1 (SnRK1) and adenosine kinase (ADK) (Figure 4). Specifically, TGMV AC2, and BCTV C2 were found to interact with and inhibit both SnRK1 and ADK (Hao et al., 2003; Wang et al., 2003). SnRK1 is a serine/threonine kinase of the SNF1/AMPK family that plays a key role in metabolism by turning off energy consuming biosynthetic pathways and turning on alternative ATP generating systems in response to nutritional, environmental, and biotic stresses that deplete ATP. This is accomplished by direct phosphorylation and inhibition of key biosynthetic enzymes as well as alteration of the transcriptome (Baena-González and Sheen, 2008; Halford and Hey, 2009; Hulsmans et al., 2016). Cellular energy charge is sensed by relative ATP/ADP/AMP levels, with AMP generally stimulating or maintaining SNF1/AMPK/SnRK1 activity. ADK is a purine nucleoside kinase that catalyzes transfer of γ-phosphate from ATP or GTP to adenosine, producing AMP. ADK is involved in adenosine salvage, which contributes to maintaining cellular energy charge by supporting the synthesis of a variety of biomolecules such as nucleotide cofactors, nucleic acids, polyamines, and enzymes involved with methyl recycling. It also plays a central role in maintaining the methyl cycle and S-adenosyl methionine (SAM)-dependent methyltransferase activity (Weretilnyk et al., 2001; Moffatt et al., 2002; Figure 4). A direct link between these two kinases has been established by the observation that SnRK1 and ADK form a cytoplasmic complex that potentially is mutually stimulatory (Mohannath et al., 2014). AMP generated by ADK is known to maintain SnRK1 activity, and SnRK1 was found to stimulate ADK by an unknown non-enzymatic mechanism. That geminiviruses appear to have evolved a dual approach for disabling metabolic responses involving these kinases leads one to conclude that they are an important aspect of plant antiviral defense.

Direct evidence for the involvement of SnRK1 in antiviral defense comes from studies which demonstrated that *N. benthamiana* plants with reduced SnRK1 activity due to expression of an antisense SnRK1 transgene display enhanced susceptibility to geminivirus infection similar to that observed upon expression of TGMV AC2 and BCTV C2 transgenes (Sunter et al., 2001; Hao et al., 2003; Mohannath et al., 2014). As SnRK1 has a plethora of cellular targets, which might be relevant to antiviral defense remains unclear, but the mRNA cap-binding proteins eukaryotic initiation factor 4E (eIF4E) and eIFiso4E were recently identified as promising candidates (Figure 4). SnRK1 has been shown to phosphorylate these essential translation initiation factors and inhibit protein synthesis (Bruns et al., 2019). This is possibly analogous to phosphorylation of eIF2α by Protein kinase R (PKR), which blocks protein synthesis in infected mammalian cells as part of the innate immune response. PKR activity, which is not found in plants, is inhibited (or its effects abrogated) by pathogenicity factors of essentially all mammalian viruses.

Interestingly, it has been reported that Arabidopsis SnRK1 can phosphorylate CaLCuV AC2 protein *in vitro.* In addition, a phosphomimic mutation in CaLCuV AC2 delayed symptom appearance in Arabidopsis and reduced viral DNA accumulation in protoplasts, suggesting that phosphorylation of AC2 by SnRK1 hinders the establishment of CaLCuV infection (Shen et al., 2014). By contrast, TGMV AC2 and BCTV C2 are not phosphorylated *in vitro* by SnRK1, and instead inhibit SnRK1 kinase activity (Wang et al., 2003). More recent work confirmed that SnRK1 can phosphorylate CaLCuV AC2 *in vitro*, but not TGMV AC2, BCTV L2, and TYLCV C2 (S. Li and D.M. Bisaro, unpublished). Consistent with this, sequence analysis revealed that the AC2/C2 proteins of some New World begomoviruses (∼20, e.g., TGMV), nearly all Old World begomoviruses examined (>100, e.g., TYLCV), and all curtoviruses (e.g., BCTV), lack a consensus SnRK1 phosphorylation site. However, the AC2 proteins of some New World begomoviruses (∼40, including CaLCuV) do in fact contain a SnRK1 consensus motif. Thus, the available evidence suggests that the SnRK1:AC2/C2 interaction is in flux: in some systems, SnRK1 may phosphorylate AC2 and reduce virus accumulation, while in most cases the AC2/C2 proteins are likely not phosphorylated and instead may inhibit SnRK1-mediated antiviral defense. That AC2/C2 may be under selection to avoid SnRK1 phosphorylation further highlights the importance of this interaction to viral pathogenesis.

Another potential consequence of inhibiting ADK relates to a possible role in cytokinin metabolism and cell cycle progression (Kwade et al., 2005). Cytokinins are N6-substituted adenine derivatives that promote cell proliferation, and ADK can phosphorylate and convert cytokinins to lower activity nucleotides (von Schwartzenberg et al., 1998). ADK may therefore modulate the relative levels of different cytokinin forms. It follows that inhibition of ADK activity by AC2/C2 could increase the pool of bioactive cytokinins necessary for plant cell cycle progression, on which geminiviruses rely for replication of their DNA genomes. Consistent with this idea, activity of a cytokinin responsive promoter was found to be increased in *adk* mutant Arabidopsis plants and in *N. benthamiana* following transient silencing of ADK expression or treatment with a pharmacological inhibitor of ADK. Similar expression changes were observed following over-expression of begomovirus AC2 and curtovirus C2. It should be noted that over-expression may not reflect the same conditions observed in a host during an actual viral infection with respect to transcript abundance, protein abundance, and/or timing of expression. However, observations that geminivirus infection increased expression of cytokinin responsive genes, and that exogenous application of cytokinin increased susceptibility to infection (Baliji et al., 2010), are consistent with AC2/C2 inhibition of ADK leading to a change in cytokinin responses and a strong indicator for ADK being a high value target for geminiviruses.

## AC2/C2 and Gene Silencing

RNA silencing refers to a set of mechanistically related, partially overlapping, and evolutionarily conserved processes including post-transcriptional gene silencing (PTGS, also known as RNA interference) and transcriptional gene silencing (TGS) (Brodersen and Voinnet, 2006; Matzke and Mosher, 2014; Pikaard and Mittelsten Scheid, 2014; Fultz et al., 2015). In plants, PTGS typically leads to siRNA-mediated degradation of mRNAs or translation inhibition in the cytoplasm. TGS is an siRNA-mediated nuclear process associated with repressive DNA and histone methylation, which is established by a pathway commonly known as RNA-directed DNA methylation (RdDM). Mechanistic details of these small RNA pathways can be found in the reviews noted above. As antiviral silencing specificity is determined by virus-derived siRNAs, viruses are both inducers and targets of the silencing response. Moreover, as siRNAs can be amplified and spread systemically throughout the plant, they can “prime” silencing-based host defenses in tissues distant from the site of primary infection, greatly enhancing their efficacy (Palaqui et al., 1997; Ratcliff et al., 1997; Molnar et al., 2010). Antiviral roles for both PTGS and TGS are well-established, and their importance is highlighted by the observation that virtually all plant viruses encode silencing suppressor proteins (Díaz-Pendón and Ding, 2008; Ruiz-Ferrer and Voinnet, 2009; Wu et al., 2010; Burgyan and Halveda, 2011). Geminiviruses, which replicate and transcribe their genes in the nucleus and export mRNAs to the cytoplasm, are targeted by both PTGS and TGS and of necessity encode proteins that suppress both pathways (Bisaro, 2006; Raja et al., 2010). These counter-defensive proteins employ multiple mechanisms to block different aspects of RNA silencing.

Post-transcriptional gene silencing was first perceived as a defense against geminiviruses with the observation that the AC2 protein of ACMV could prevent silencing of a green fluorescent protein (GFP) transgene in *N. benthamiana* plants (Voinnet et al., 1999). AC2 was subsequently found to suppress PTGS by multiple mechanisms. One, referred to here as transcription-dependent suppression, involves transactivation of host genes that appear to encode endogenous negative regulators of RNA silencing, including Werner exonuclease-like 1 (*WEL1*) and regulator of gene silencing calmodulin-like protein (*rgs-CaM*) (Trinks et al., 2005; Chung et al., 2014). A second mechanism, referred to as transcription-independent suppression, is shared by AC2 and BCTV C2 which, unlike AC2, is not a transcriptional activator. This mechanism correlates with the ability of AC2 and C2 to interact with and inactivate ADK (Wang et al., 2003, 2005). Interestingly, while both AC2 and C2 can inhibit the establishment of PTGS, only AC2 can block the systemic spread of silencing. That AC2 lacking its transcription activation domain is likewise unable to prevent systemic spread indicates that suppression of this aspect of silencing is transcription-dependent (Jackel et al., 2015). Because silencing spread is a crucial feature of antiviral defense, it is possible that another BCTV protein might be involved in preventing the production and/or trafficking of mobile siRNAs.

Transcriptional gene silencing was first implicated as a defense against geminiviruses with the observation that viral replication in transfected protoplasts is greatly reduced when the inoculum DNA is methylated (Brough et al., 1992). Later studies employing a variety of Arabidopsis mutants lacking components of the RdDM pathway definitively established that viral chromatin methylation is a potent epigenetic defense against geminiviruses (Raja et al., 2008, 2014; Jackel et al., 2016). Repressed viral chromatin is covalently marked with cytosine methylation and histone H3 lysine 9 dimethylation (H3K9me2), both of which are hallmarks of constitutive heterochromatin that are also found on silenced endogenous transposable elements. In addition, viral chromatin containing epigenetic marks indicative of active viral gene expression (including H3K9 acetylation and H3K4me3) coexist with repressed viral chromatin in infected plants, and the equilibrium between them dictates the outcome of infection. A preponderance of repressed chromatin favors symptom remission and host recovery from infection (Raja et al., 2008, 2014; Ceniceros-Ojeda et al., 2016; Jackel et al., 2016; Coursey et al., 2018b). Repressed viral chromatin is highly compacted relative to active chromatin, and increased physical compaction correlates with reduced viral gene expression (Ceniceros-Ojeda et al., 2016; Coursey et al., 2018b). However, the relationship between active and repressed chromatin may prove more complex than initially realized. EMSY-like 1 (EML1), a histone reader protein that binds a mark often present on active chromatin (H3K36 methylation), was recently found to suppress geminivirus infection. Further, EML1 was shown to diminish viral gene expression by inhibiting the association of RNA polymerase II (Pol II) with viral chromatin (Coursey et al., 2018a). Thus, EML1 may bind viral chromatin marked as active and promote changes that render it less accessible to the cellular transcription machinery.

TGMV and CaLCuV AC2 have been shown to suppress and reverse viral chromatin methylation and TGS by both transcription-dependent and -independent means, while BCTV C2 is again limited to the latter mechanism (Buchmann et al., 2009; Jackel et al., 2015). While transcription-dependent mechanisms are not yet defined, AC2 binds and inhibits the histone methyltransferase responsible for writing repressive H3K9me2, a crucial RdDM pathway component (Castillo-González et al., 2015). Transcription-independent reversal of TGS also correlates with methyl cycle interference due to inhibition of ADK (Wang et al., 2003; Buchmann et al., 2009; Figure 4). By phosphorylating adenosine, ADK promotes flux through the methyl cycle that generates SAM, a methyl group donor and essential methyltransferase cofactor (Moffatt et al., 2002). The importance of the methyl cycle for defense against geminiviruses is underscored by the number of pathway enzymes targeted by viral proteins. In addition to ADK targeted by AC2 and BCTV C2, BSCTV C2 inhibits methylation by stabilizing SAM decarboxylase (SAMDC), presumably increasing levels of decarboxylated SAM (dcSAM) at the expense of SAM (Zhang et al., 2011). It should be pointed out that the cellular levels of SAM or dsSAM were not measured directly. Interestingly, the βC1 protein encoded by the TYLCCNV satellite DNAβ (TYLCCNB) interferes with SAM synthesis by interacting with and inhibiting S-adenosyl homocysteine hydrolase (SAHH) (Yang et al., 2011). In this case, C2 encoded by the TYLCCNV helper virus appears to have lost the ability to suppress TGS, relying instead on βC1 to provide this critical function. Yet another geminivirus protein, C4 encoded by *Cotton leaf curl Multan virus* (CLCuMuV), suppresses TGS and PTGS by interacting with SAM synthetase (Ismayil et al., 2018). Clearly, methyl cycle inhibition is a common strategy of begomoviruses and curtoviruses.

## AC2/C2, Autophagy and rgs-CaM

**FIGURE 5:**
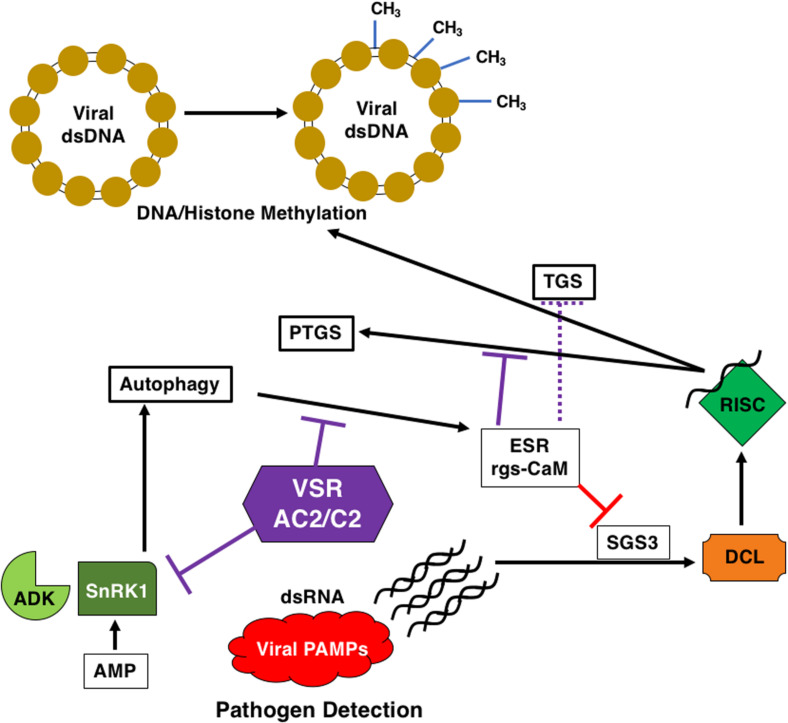
AC2/C2 and Autophagy. AC2/C2 modulate the autophagic pathway to facilitate infection and inhibit antiviral defense pathways. AC2/C2 stabilize endogenous suppressors of RNA silencing to inhibit TGS and PTGS. The following cellular components are shown: ADK, Adenosine Kinase; SnRK1, SNF1-Related Kinase 1; PTGS, Post-Transcriptional Gene Silencing; ESR, Endogenous Suppressor of RNA Silencing; rgs-CaM, Regulator of Gene Silencing Calmodulin-like Protein; TGS, Transcriptional Gene Silencing; SGS3, Suppressor of Gene Silencing 3; DCL, Dicer Like; RISC, RNA-Induced Silencing Complexes; VSR, Viral Suppressor of RNA Silencing; AC2/C2, geminivirus virulence factors; CH_3_, methylated DNA in the viral dsDNA minichromosome. Black arrows represent reactions and/or responses that are induced. Red lines indicate inhibition due to the ESR. Purple lines indicate inhibition of ADK and SnRK1 and rgs-CaM through interaction with AC2 and/or C2 proteins.

Recent research has implied a link between autophagy, infection and RNA silencing (Figure 5). Autophagy is a cell-based self-degradative process important for energy balance during critical times in development and in response to different stresses, including nutrient deprivation and viral infection (Zhou et al., 2014). RNA silencing as mentioned earlier is part of the innate immune response in plants, and it has been shown that components of the RNA silencing pathway are targeted for degradation by the host autophagic pathway (Li et al., 2014, 2017). Viral RNA silencing suppressors are also targeted by the autophagic pathway, potentially through the action of rgs-CaM (regulator of gene silencing–calmodulin-like) (Anandalakshmi et al., 2000; Nakahara et al., 2012). It has been proposed that rgs-CaM acts as an endogenous negative regulator of RNA silencing, ensuring that this arm of the immune system is inactive in the absence of a viral infection. The HC-Pro protein encoded by the *Tobacco etch virus* (TEV) is a suppressor of PTGS and has been shown to interact with rgs-CaM from *Nicotiana tabacum* (Nt-rgsCaM), and to increase the levels of Nt-rgsCaM (Anandalakshmi et al., 2000). It was later discovered using tobacco cells that rgs-CaM is capable of interacting with other viral RNA silencing suppressors (RSS), including HC-Pro encoded by the potyvirus *Turnip mosaic virus* (TuMV) and *Cucumber mosaic virus* (CMV) 2b protein (Nakahara et al., 2012). This was further extended by the observation that rgsCaM RNA levels are increased *N. benthamiana* infected with the begomoviruses CaLCuV and TGMV, and with the curtovirus BCTV (Chung et al., 2014). This increase was recapitulated when TGMV AC2 was over-expressed in plants (Chung et al., 2014). Further, transcriptomic studies revealed that rgs-CaM is up-regulated in response to HC-Pro, P25 from *Potato virus X* (PVX), and ACMV AC2 (Jada et al., 2013). Binding of rgs-CaM to CMV 2b appears to be mediated through an arginine rich region of the viral protein. Interestingly, the AC2 protein of begomoviruses has a conserved basic region containing a stretch of arginine residues (Figure 2). TGMV AC2 is able to interact with rgs-CaM (Chung et al., 2014), although it is not known whether C2 proteins are capable of interacting with rgs-CaM. It is interesting to note at this point that the βC1 protein encoded by the betasatellite TYLCCNB has also been shown to upregulate rgs-CaM in *N. benthamiana* (Nbrgs-CaM), resulting in suppression of RNA silencing through repression of RNA dependent RNA polymerase 6 (RDR6) expression (Li et al., 2014, 2017). Additional studies determined that Nbrgs-CaM is able to interact with and induce autophagic degradation of Suppressor of Gene Silencing 3 (SGS3), a cofactor of RDR6 in PTGS (Li et al., 2017). While the βC1 protein sequence is unrelated to AC2, they both function as silencing suppressors and so autophagic degradation may be a general antiviral response targeting viral suppressors. Interestingly, both rgs-CaM and viral suppressors of PTGS are likely degraded by autophagy-like protein degradation (ALPD) immediately after they form a complex (Nakahara et al., 2012). However, for geminiviruses interaction with AC2 results in a different outcome. While rgs-CaM is able to self-interact in the cytoplasm, AC2 sequesters rgs-CaM to localized regions of the nucleus (Chung et al., 2014). The apparent difference in AC2 interaction outcome as compared to viral suppressors from previous studies could be a consequence of the DNA genome of geminiviruses, or that rgs-CaM inhibits the ability of other suppressors to bind siRNAs (Nakahara et al., 2012). This may well be the case given that TGMV AL2 does not bind siRNAs, even under conditions that support binding by the *Tomato bushy stunt virus* P19 suppressor (Wang et al., 2005). Although we do not know at this time whether nuclear relocalization of rgs-CaM by AC2 has any role in silencing suppression, another interesting possibility relates to the function of AC2 in suppression of TGS. Given that nucleolus-associated Cajal bodies in plants are possible sites for biogenesis of siRNAs that guide TGS RISC complexes to chromatin (Pontes and Pikaard, 2008), it is tempting to speculate that sequestration of rgsCaM by AC2 could impact the ability of the host to generate siRNAs for silencing in general, or more specifically for TGS. The potential importance of rgs-CaM to host defense against geminivirus infection is highlighted by observations that over-expression in *N. benthamiana* plants leads to enhanced susceptibility to TGMV infection, while *Arabidopsis* plants containing an rgs-CaM T-DNA insertion mutation is are less susceptible to infection by CaLCuV and BCTV (Chung et al., 2014). The role of autophagy in potentially limiting geminivirus infection, the potential inhibition through the function of AC2/C2 and the link to RNA silencing is based on a few limited studies using over-expression strategies, and so additional work is needed to confirm whether autophagy represents a true anti-viral defense against geminiviruses.

Despite the differences, these results are consistent with the involvement of endogenous silencing suppressors in the mechanism of action of viral RNA silencing suppression. Referring back to the zigzag model for plant immunity (Jones and Dangl, 2006), the dsRNA trigger for RNAi could well be regarded as a viral PAMP and RNA silencing considered to be a facet of PAMP-triggered immunity (Nakahara et al., 2012; Figure 5). The RNA silencing defense is then countered by viral suppressor proteins like AC2, which can function at different points in the pathway to decrease the availability of siRNAs for the silencing machinery (Bisaro, 2006; Raja et al., 2010). Thus, viral suppressors can be regarded as effectors that facilitate viral infection and replication in plants (Nakahara et al., 2012). As a possible counter-defense, rgs-CaM may be able to recognize AC2 and subsequently target the protein for degradation, but geminiviruses may have evolved different strategies to evade this defense. AC2 from TGMV appears to sequester rgs-CaM in the nucleus, whereas the TYLCCNB βC1 protein promotes degradation of SGS3.

## The Geminivirus Replication Cycle Is Regulated by Temporally Controlled Gene Expression

**FIGURE 6:**
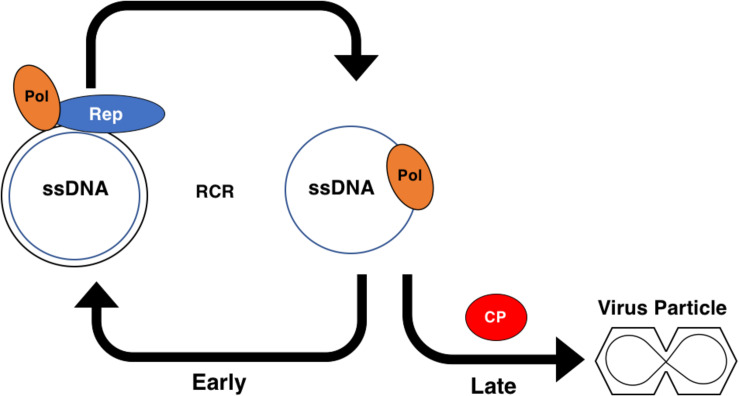
Role of the Geminivirus CP Protein in Rolling Circle Replication. Early in the infection cycle single-stranded (ss)DNA associates with host DNA polymerases (Pol) and is converted to a double-stranded (ds)DNA intermediate. The dsDNA intermediate associates with the viral Rep protein and Pol to serve as a template for amplification via rolling circle replication (RCR). The dsDNA intermediate also serves as a template for transcription and produces the viral coat protein (CP) transcript late in the infection cycle. CP sequesters the viral ssDNA into particles, preventing the ssDNA from participating in RCR.

In addition to the extensive role that AC2 plays in suppression of host immune responses, a major function of AC2 is to regulate expression of the late viral genes, CP and NSP. Early in the infection process, after the viral genomic ssDNA has entered the nucleus, host polymerases use viral DNA as template for complementary strand synthesis to generate dsDNA RF intermediates, which subsequently associate with histones to form minichromosomes (Figure 6). Viral minichromosomes serve as template for both transcription and rolling circle replication (RCR). Minichromosomes of bipartite begomoviruses associate with 11 or 12 nucleosomes in two defined structures with open gaps that correlate with promoter structures and the origin of replication in both DNA A and DNA B (Pilartz and Jeske, 2003). One of the nucleosome free regions spans the IR, which contains the origin of replication and divergent promoters for complementary and viral sense transcription. A large complementary sense transcript encodes the early viral genes (Rep and AC3) that promote viral replication and production of genomic ssDNA by RCR. Early in infection, newly synthesized ssDNA is converted to dsDNA to amplify RF intermediates. Subsequent binding of Rep within the IR down-regulates its own expression (Sunter et al., 1993; Eagle et al., 1994), which enables expression from a downstream promoter that generates a transcript capable of expressing AC2. The downstream promoter appears to correlate with the second nucleosome-free region on minichromosomes. AC2 in turn activates expression of late genes (CP and NSP) from the virion sense promoter in the IR. Expression of late genes promotes virus spread and encapsidation of genomic ssDNA (Sunter and Bisaro, 1992). The presence of CP is critical for ssDNA accumulation during RCR. Thus, AC2 is critical for regulating the timing of CP expression to ensure that ssDNA is converted to dsDNA early during an infection and is sequestered late in the infection (Figure 6).

## AC2 and *CP* Promoter Regulation

**FIGURE 7:**
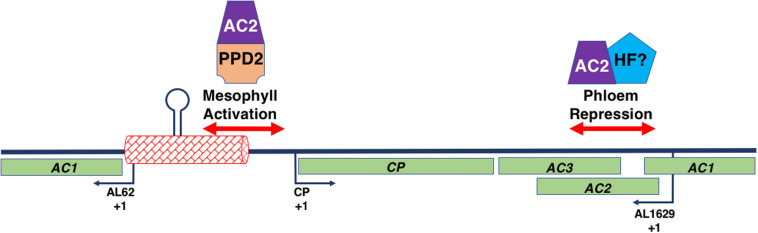
Interaction of the Begomovirus Genome with AC2 and Host Factors. The figure represents a double stranded begomovirus DNA A genome in linear form. The complete CP, AC2, and AC3 open reading frames are shown (light green boxes), along with the AC1 ORF, which is split into two. The transcription initiation sites (+1) for the viral (CP) and complementary (AL62 and AL1629) sense transcripts are indicated by the black arrows. The intergenic region (red box) contains the replication origin including the hairpin and its invariant loop sequence (lollipop). Regions of the begomovirus genome involved in AC2-mediated activation in mesophyll and AC2-mediated de-repression in phloem tissue are shown (double-red arrows). An AC2-PPD2 complex binds sequences with the *CP* promoter involved in AC2-mediated activation. Whether AC2 interacts with a host factor (HF) to mediate de-repression is currently unknown.

AC2-mediated regulation of the *CP* promoter in begomoviruses occurs in all tissues, however the mechanism by which expression is controlled is different in different cell types (Sunter and Bisaro, 1997). It has been determined that AC2 activates the *CP* promoter in mesophyll cells but acts to derepress and activate the *CP* promoter in phloem cells (Sunter and Bisaro, 1997). This is mediated through independent sequences located in two different regions of the viral genome (Figure 7). AC2-dependent *CP* promoter activation in both phloem and mesophyll cells is mediated through sequences located proximal to the transcription start site of the *CP* gene, but downstream of the conserved stem-loop structure important for the initiation of replication (Sunter and Bisaro, 1997, Bisaro, 2003; Lacatus and Sunter, 2008). In TGMV, a second element located within a region 590 bp downstream of the CP coding region, within the AC2 and AC3 coding sequences, is necessary for repression of *CP* promoter activity in phloem cells (Sunter and Bisaro, 1997). Repression of CaLCuV *CP* promoter activity in phloem cells is mediated by sequences within 340 bp downstream of the CP coding region, again within the AC2 and AC3 coding sequences (Lacatus and Sunter, 2008). AC2 is also required for activation of the *NSP* promoter (Sunter and Bisaro, 1992) and is dependent on sequences within 144 bp upstream of the NSP transcription start site (Berger and Sunter, 2013). Although the *NSP* promoter appears to exhibit AC2-independent expression in vascular tissue, similar to the CaLCuV and TGMV *CP* promoters (Berger and Sunter, 2013), we do not currently know whether the *NSP* promoter is regulated by independent mechanisms in different tissue types. By comparison, we have previously noted that the C2 protein in curtoviruses appears to lack the ability to activate transcription (Sunter et al., 1994). Further, analysis of the BCTV-SpCT *CP* promoter in transgenic *N. benthamiana* plants demonstrated it is active in the absence of any viral proteins (Rao and Sunter, 2012). Given the assumed necessity to control CP production as outlined for begomoviruses, the *CP* promoter is expected to be repressed in phloem cells, although this has currently not been tested. Therefore, we speculate that late in infection it is possible that C2 acts to derepress the promoter thereby producing CP at the appropriate time.

The interaction of AC2 with two independent sequences within the begomovirus genome, in conjunction with the observation that these sequences appear to bind different nuclear factors, suggests that AC2 is capable of interacting with different components of the host transcription machinery proteins to regulate the viral *CP* promoter (Sunter and Bisaro, 1997; Lacatus and Sunter, 2008; Figure 7). As AC2 does not bind dsDNA in a sequence-specific manner (Hartitz et al., 1999), it was assumed that AC2 is directed to responsive promoters through protein-protein interactions with cellular factors (Sunter and Bisaro, 2003). This was confirmed when a genetic screen identified the Arabidopsis PEAPOD2 (PPD2) protein, which is also known as TIFY4B, which specifically binds to sequences within the TGMV and CaLCuV *CP* promoters that mediate AC2-dependent promoter activation (Sunter and Bisaro, 2003; Lacatus and Sunter, 2009). The idea that AC2 is targeted to the *CP* promoter through interaction with PPD2, leading to activation of *CP* gene expression, is supported by evidence that PPD2 is also able to bind sequences necessary for AC2-mediated activation of the TGMV *NSP* promoter (Berger and Sunter, 2013), but not with sequences required for AC2-mediated derepression in phloem (Lacatus and Sunter, 2009). Additional evidence that AC2 is targeted to responsive promoters by PPD2 is provided by results which demonstrate that TGMV AC2 and PPD2 are able to form a complex on sequences containing the *CP* promoter, and that PPD2 localizes to the nucleus but is unable to activate transcription directly (Lacatus and Sunter, 2009). The ability of AC2 proteins from different viruses to transactivate the TGMV *CP* promoter suggests that begomovirus AC2 gene products function through interactions with common host proteins (Sunter et al., 1994). This is consistent with the observation that AC2 proteins from different begomoviruses are able to directly interact with PPD2 (Lacatus and Sunter, 2009).

## Impact of AC2 on the Host Transcriptome: Computational Analysis

If AC2 is targeted to the *CP* and *NSP* promoters by a host factor(s), then it is expected that AC2 could also have widespread impact on the host transcriptome. Several large-scale microarray studies have been conducted to survey changes in the host transcriptome induced by the AC2 protein or its homologs (Trinks et al., 2005; Caracuel et al., 2012; Soitamo et al., 2012; Liu et al., 2014). Applying a stringent statistical criterion to a study using AC2 of ACMV and *Mungbean yellow mosaic virus-Vigna* (MYMV), Affymetrix GeneChips (ATH1), and transient expression assays with *Arabidopsis* protoplasts, 55 genes were found to be up-regulated >2-fold by MYMV AC2 (Trinks et al., 2005). Of these 55 genes, 30 were also induced >2-fold by ACMV AC2. With a less stringent criterion, the number of genes upregulated by ACMV increased to 162, of which 139 were also induced by MYMV AC2, including six cold-regulated genes. A second study using Agilent’s microarray platform examined expression in transgenic tobacco plants expressing ACMV AC2, and identified a total of 1369 differentially expressed genes in leaves and flowers. Examples of the types of processes whose genes were found to be up-regulated were those related to stress, cell wall modification, and signaling. By contrast, processes associated with genes that were down-regulated were those related to translation, photosynthesis, and transcription (Soitamo et al., 2012). A comparison of the transcriptomic changes in transgenic Arabidopsis plants expressing C2 from the monopartite begomovirus TYLCSV, and its curtovirus counterpart, C2 from BCTV, found that the BCTV C2 up-regulates 444 genes and down-regulates 154 (Caracuel et al., 2012). Among those genes up-regulated, 15 were related to the cell-cycle and 9 were associated with DNA packaging. In contrast, stress response genes are over-represented in both up- and down-regulated genes. However, there was minimal overlap between genes differentially regulated by TYLCSV C2 and BCTV C2 (Caracuel et al., 2012). Although these studies were performed using over-expression of the viral proteins, it is important to validate the differential expression of identified candidate genes during an actual viral infection.

**FIGURE 8:**
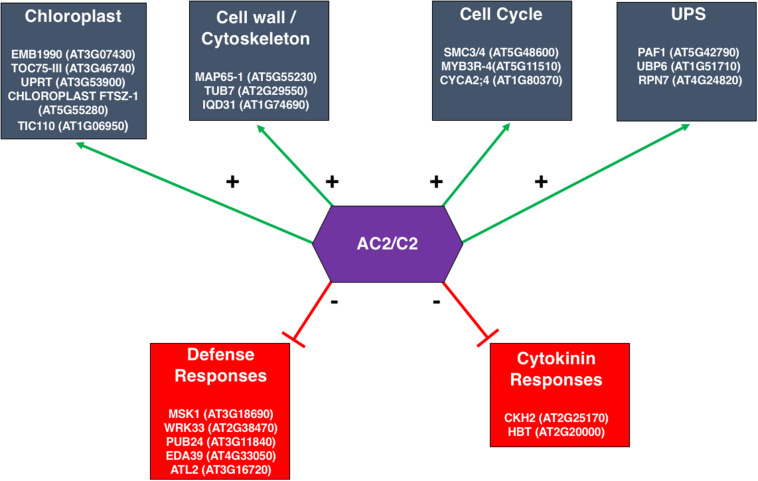
Sub-networks of Genes Differentially Expressed in the Arabidopsis Genome in Response to the Transcription Function of the CaLCuV AC2 Protein. The response of Arabidopsis transcriptome to full-length CaLCuV AC2 or AC2 with a deletion of the transcriptional activation domain, revealed significant alterations in gene expression that could be attributed to the transcription function of AC2. A network-based analysis identified core gene groups that were up- or down-regulated in response to full length AC2 at 1 and 2 dpi, (Liu et al., 2014). Examples of genes, including gene ID, within these networks are given within each of the boxes shown. Downregulated groups are shown in red, and upregulated groups are in blue.

A dilemma frequently faced by genome-wide expression studies is that, due to the problem of multiple hypothesis testing and limited statistical power, very few genes can be selected with stringent statistical significance. On the other hand, with reduced statistical rigor, many more genes can be selected but it is difficult to determine which genes are indeed differentially expressed in response to a given treatment. To address this problem, Liu et al. (2014) developed a network-based analysis to identify core gene groups responding to AC2 expression in Arabidopsis using a whole plant infusion assay with either the full-length CaLCuV AC2 or a truncated AC2 lacking the C-terminal transcriptional activation domain. Host genes that were differentially expressed due to full-length or truncated AC2 were overlaid to a whole-genome gene co-expression network constructed from more than 1300 microarrays (Ruan et al., 2011). Although hundreds of genes were identified as differentially regulated by full length AC2 but not truncated AC2, most of them were not connected to each other in the network, reflecting the diverse functional processes induced by AC2. Interestingly, a small fraction of the genes appears to be tightly linked to each other, resulting in dense sub-networks that may represent core functional groups co-regulated by the transcriptional activation function of AC2. For example, of the 214 unique genes that were up-regulated in response to full length AC2 at one day post infusion (dpi), five subnetworks were identified, each consisting of between four to eight highly connected genes. Two of these subnetworks contain genes encoding complexes involved in protein import into chloroplasts, which are of potential relevance for geminivirus infections (Krenz et al., 2012). Another sub-network consists of genes associated with the cell wall and/or cytoskeleton (Figure 8), consistent with previous findings using ACMV AC2 (Soitamo et al., 2012). This supports the hypothesis that AC2 may induce host genes that are important for cell-to-cell and long-distance movement of the virus. Of the six sub-networks up-regulated by full length AC2 at 2 dpi, one consists of five highly inter-connected genes having functions related to the cell cycle (Figure 8), in agreement with microarray results using BCTV C2 (Caracuel et al., 2012). On the other hand, down-regulated genes form a dense sub-network of genes involved in defense responses to pathogen infection (Figure 8). Another sub-network of down-regulated genes included cytokinin-hypersensitive 2 and Hobbit. This down-regulation may hint at a potential mechanism whereby AC2 interferes with progression of cell differentiation, shifting the balance in favor of cell proliferation thereby promoting viral replication (Figure 8). Many of these genes would not have been thought of as functioning in a network using standard statistical approaches, and therefore a network-based analysis can reveal highly connected genes in co-regulated gene networks that are potentially targeted by geminiviruses during infection.

It is quite clear from several studies that geminiviruses manipulate the host transcriptome, and that some critical networks appear to be high-value targets of the AC2/C2 protein. However, substantial work needs to be done to determine the consequences of these changes in host gene expression for viral pathogenesis.

## Future Perspectives

It is clear that the AC2/C2 proteins encoded by begomoviruses are multifunctional and have critical roles in both suppression of host immune responses and in regulation of the *CP* promoter. While the information gathered to date is extensive and has increased our understanding of the role of AC2 in geminivirus pathogenesis, there are several aspects with respect to AC2 that are currently unknown and are, or we argue should be, under investigation. First, the mechanism(s) by which AC2/C2 interact to disable the UPS are not understood, nor is it clear that we have discovered all of the defense-related pathways that are targeted by these viral proteins. As an example, plant hormones have significant roles in plant–pathogen interactions (Alazem and Lin, 2014), and the known interactions between these hormones along with the interaction of AC2/C2 with the cytokinin, SA, and JA pathways, leads us to believe that geminiviruses may also perturb other hormone pathways as well. Second, additional information is needed on how AC2 reverses TGS at the IR. Third, we do not know whether there are additional host factors that interact with the begomovirus *CP* promoter to mediate activation in mesophyll, and the identity of the host factor(s) that mediates repression in phloem cells is unknown. Fourth, the mechanism by which geminiviruses switch from early to late gene expression requires further study. For example, are there different populations of dsDNA templates for replication and transcription, or is a single population used for both? Lastly, how are the different functions of AC2 regulated? Consider that TGMV AC2 protein can interact with ADK, SnRK1, rgsCaM, and PPD2. We know that AC2 is found in both phosphorylated and non-phosphorylated forms in plant cells (Wang et al., 2003), and that AC2 can form a homo-dimer and higher-order multimers, which is consistent with a role in transcriptional activation (Yang et al., 2007). So, are there different functions associated with phosphorylated and non-phosphorylated forms and/or multimers? Answers to these and other questions will be valuable in extending our knowledge of AC2 function, which may ultimately lead to new ideas on how to effectively combat this devastating group of viruses.

## Author Contributions

GS: writing and editing entire manuscript, figures, and submission. JG: writing, draft of AC2 and HR, UPS, sulfur and transcription section. ER: writing, draft of metabolism, silencing section, and figures. LL and JR: writing, drafts computational section. DB: writing and editing the entire manuscript.

## Conflict of Interest

The authors declare that the research was conducted in the absence of any commercial or financial relationships that could be construed as a potential conflict of interest.
